# Challenges Associated with the Identification of Autism Spectrum Condition Symptomatology in Girls: A Grounded Theory Lite Approach

**DOI:** 10.1192/j.eurpsy.2024.968

**Published:** 2024-08-27

**Authors:** N. Dolović

**Affiliations:** Angerona, educational and rehabilitation private practice, Čakovec, Croatia

## Abstract

**Introduction:**

Female Autism Spectrum Conditions (FASC) often go without a proper diagnosis, receive misdiagnoses, or are diagnosed late in life compared to males.These circumstances can lead to negative consequences in their overall health, emotional well-being, educational attainment, job opportunities, and independence. There is a growing body of literature highlighting distinctions between females and males in the context of autism. Regrettably, these insights are not effectively making their way into practical applications. While shifting entrenched perspectives among practitioners is a gradual process, there is an immediate and pressing need for change in this regard. Numerous misconceptions persist regarding the presentation of FASC, hindering the recognition of its diverse effects based on an individual’s cis-gender identity or other factors.

**Objectives:**

The purpose of this study is to identify key challenges associated with the identification of ASC symptomatology in girls, with the goal of informing future research and clinical practice.

**Methods:**

Using constant comparative analysis applicable to grounded theory lite with an inductive approach, this study employs an interpretative research methodology with a focus on generating theory from qualitative data, albeit with certain shortcuts or less resource-intensive steps. Data were collected through interviews providing insights into their experiences, behaviors, and developmental history, observations enabling to capture real-time behavioral and communicative patterns., and notes during first and initial developmental assessment, as well as using ADOS-II with some participants (according to referrals and parents’ decision), in the period of 3 years (2020-2023) from 25 girls age 18 months to 15 years and their mothers, and occasionally both parents.

**Results:**

Preliminary findings indicate a complex interplay of behavioral, communicative, and social challenges in these girls, shedding light on potentially distinctive patterns of symptom expression in comparison to boys. Furthermore, barriers hindering parental involvement in the diagnostic process have also been identified. This study holds significant importance as it may inform future research efforts aimed at addressing these challenges that currently impede clinicians in the early identification of FASC, which manifests quite differently in girls compared to boys.

**Conclusions:**

Taking into account certain study limitations, the significance of this research lies in its capacity to influence future research initiatives. By illuminating the obstacles that hinder clinicians in the early detection of FASC, which manifest distinctively in girls compared to boys, it emphasizes the pressing need to address these challenges. This, in turn, enhances early detection and support systems for FASC, ultimately contributing to their well-being and quality of life.

**Disclosure of Interest:**

None Declared

